# State-of-the-Art Mixed Matrix Membranes (MMMs)

**DOI:** 10.3390/membranes12030294

**Published:** 2022-03-04

**Authors:** Harsh Vardhan, Francis Verpoort, Wenwen He

**Affiliations:** 1 Department of Chemistry and Fermentation Sciences, Appalachian State University, 525 Rivers Street, Boone, NC 28608, USA; 2 State Key Laboratory of Advanced Technology for Materials Synthesis and Processing, Wuhan University of Technology, Wuhan 430070, China; 3 National Research Tomsk Polytechnic University, Lenin Avenue 30, 634050 Tomsk, Russia; 4 School of Chemistry and Life Science, Advanced Institute of Materials Science, Changchun University of Technology, Changchun 130012, China; heww@ccut.edu.cn

The performance of most polymer membranes suffers from the trade-off relationship between permeability and selectivity. To expand the applications of polymeric membranes, the advancement in novel membrane fabrication technology is of profound importance. The fragility, high cost, and processability of inorganic membranes hinder large-scale industrial applications, including gas separation. The amalgamation of the benefits of both inorganic and polymeric materials to fabricate mixed matrix membranes (MMMs) offers an avenue to overcome the shortcomings of both membrane types. Defect-free, robust MMMs balance the trade-off relationship between permeability and selectivity. Inorganic filler-based MMMs, organic filler-based MMMs, hybrid filler-based MMMs, and bio-filler-based MMMs, offer better performance, fouling, permeate quality, and longevity solutions, and have attracted considerable attention in natural gas purification, wastewater treatment, and oil exploitation. Pioneering work in this domain has corroborated the MMMs tool as a dependable platform to disclose potential challenges.

This Membranes Special Issue (S.I.), titled “State-of-the-art mixed matrix membranes (MMMs)”, highlights the latest results of scientific research on the fabrication of novel MMMs systems and sheds light on the grafting time to establish interaction between fillers and polymers. Furthermore, the role of membrane systems in industrial applications, such as wastewater treatment, is covered. Five papers were published covering the fabrication and performance of MMMs in gas separation and wastewater treatment. A summary of the research articles is presented here.

Martinez-Tirado et al. [1] studied the formation of novel MMMs, using Matrimid (M), polysulfone (PSF) or polyphenylene oxide (PPO) as the continuous phase and porous biphenyl-based knitting aryl polymer (K_2_Ph), prepared with the Scholl method and Friedel–Crafts reaction as a filler. The polymer matrices were subjected to a controllable addition of K_2_Ph, which led to M@K_2_Ph-10%, M@K_2_Ph-20%, PSF@K_2_Ph-20%, and PPO@K_2_Ph-5% membrane systems. Scanning electron microscopy (SEM) highlighted good compatibility between the matrices and the filler; however, the K_2_Ph slightly decreased the thermal stability of all the matrices. The authors found that the permeability and diffusion coefficient follows the trend P(H_2_) > P(CO_2_) > P(O_2_) > P(C_2_H_4_) > P(N_2_) ≥ P(CH_4_), and D(H_2_) > D(O_2_) > D(CO_2_) > D(N_2_) > D(CH_4_) ≥ D(C_2_H_4_), respectively, for all MMMs. This study suggests the role of K2Ph as a filler, M@K_2_Ph-20%, increasing the permeability coefficients of different gases by a factor of 2–3; however, this was around one-and-a-half times for matrix PSF. More importantly, novel MMMs have the highest H_2_/CH_4_ and H_2_/C_2_H_4_ selectivities, making them viable for the recovery and purification of hydrogen from petrochemical industries.

Hong et al. [2] illustrated the fabrication of porous membranes with a water pressure treatment, using cellulose acetate (C.A.) and propylene glycol as matrix and additives, respectively. The membrane (1:0.2 C.A./propylene glycol) pores can be generated and controlled through water pressure due to plasticization. The authors established the interaction of the C.A. and propylene glycol via Fourier transform infrared (FTIR) spectroscopy, as the ether peaks and carbonyl peaks shifted from 1031 cm^−1^ to 1039 cm^−1^, and 1737 cm^−1^ to 1745 cm^−1^, respectively. Furthermore, with high thermal stability, the 1:0.2 CA/propylene glycol membrane exhibited a water flux of 14.87 L/m^2^ h. This investigation highlights an alternative to metal salt as an additive to fabricate porous membranes.

To address fouling in membrane-based technology processes, Wibisono et al. [3] employed cocoa pod husk extract (CPHE) in phenolic nanoparticle form, in varying concentrations of 0.5 wt%, 0.75 wt%, and 1.0 wt%, into cellulose acetate polymer. The dry–wet phase inversion method used dimethylformamide (DMF) and dimethylacetamide (DMAc) to fabricate a CPHE phenolic nanopowder/C.A. mixed matrix membrane, with an average thickness and contact angle of 0.09–0.11 mm and 40°–60°, respectively. More importantly, for membranes prepared in DMF, the tensile strength value increased with the addition of 0.5 wt% CPHE and decreased with a higher composition of additives. However, for membranes prepared in DMAc, the membrane tensile strength increases until the addition of 0.75 wt% CPHE. The authors conclude that 1.0 wt% CPHE/CA membranes prepared with DMAc possessed the highest water flux (2.34 Lm^−2^ h^−1^). In addition, the increase in the amount of CPHE nanopowder reduces the amount of Escherichia coli to the C.A. membrane by 70.8% (DMAc), and 90.5% (DMF), addressing biofouling to expand the membrane applications.

The influence of the grafting time (30, 60, and 90 mins) of the TiO_2_ nanocomposite on the properties and performance of the bentonite membranes, toward oil rejection and pure water flux, is studied by Mohamad Esham et al. [4]. The authors used sol dip-coating and the sol-gel method to optimize the grafting time (30, 60, and 90 mins) of TiO_2_ nanoparticles on a bentonite membrane. FTIR analysis of Ti-Ben 30, Ti-Ben 60, and Ti-Ben 90-coated membranes exhibited Ti-O, and Ti-OH peaks at 1000 cm^−1^ and 1620 cm^−1^, respectively. The peak intensity increases as grafting time increases, highlighting the penetration of nanoparticles into the membrane. The authors found that Ti-Ben 60 displayed an outstanding pure water flux performance, with a 67% increment, compared to a pristine bentonite membrane. Furthermore, the TiO_2_-coated membrane exhibited an oil rejection performance of 98.5%. This study illustrates the pivotal role of the surface coating of bentonite membranes with a TiO_2_ nanoparticle, which has great potential for industrial wastewater solutions, particularly in terms of oil removal.

Nazri et al. [5] illustrated the fabrication of novel microcrystalline cellulose (MCC)-incorporated polyethersulfone (PES) membranes, through a phase inversion approach, using a LiCl/DMAc co-solvent. Compared to the pristine PES membrane, the composite membrane with varying MCC concentration (1 wt%, 3 wt%, 5 wt%) has larger surface sponge-like pores, hydrophilicity (abundant hydroxyl groups), and viscosity. The FTIR spectrum of 1 wt% MCC, 3 wt% MCC, and 5 wt% MCC incorporated PES membrane exhibited significant changes at the peak region, 3398 cm^−1^, 2898 cm^−1^, and 1647 cm^−1^, representing -OH groups, -CH_2_ groups, and -H_2_O, respectively. With a 3 wt% MCC loading composite membrane, the authors found pure water flux (PWH) and high humic acid (HA) rejection of 51.34 and 96.14%, respectively, which is 20 times higher and a 6.8% increase, as compared to the pristine PES membrane. The authors propose an alternate and competitive approach to MMMs loaded with graphene oxide (GO) and cellulose nanocrystal (CNC) additives.

Taken together, the articles published in this Special Issue will refurbish and further develop our current knowledge on MMMs. These studies with various technical approaches, such as SEM, will provide an insight into the fabrication of MMMs underlying the role of matrices and fillers, and grafting time.
